# Applied Machine Learning for Stochastic Local Volatility Calibration

**DOI:** 10.3389/frai.2019.00004

**Published:** 2019-05-17

**Authors:** Jürgen Hakala

**Affiliations:** Quantitative Modeling Department, Leonteq Securities AG, Zurich, Switzerland

**Keywords:** radial basis functions, machine learning, local stochastic volatility, derivatives pricing, finance

## Abstract

Stochastic volatility models are a popular choice to price and risk–manage financial derivatives on equity and foreign exchange. For the calibration of stochastic local volatility models a crucial step is the estimation of the expectated variance conditional on the realized spot. The spot is given by the model dynamics. Here we suggest to use methods from machine learning to improve the estimation process. We show examples from foreign exchange.

## 1. Introduction

For derivatives pricing a major breakthrough was achieved with the risk-neutral valuation principle (Black and Scholes, 1973). Initially the models assumed a deterministic, state-independent volatility of the underlying price process. For many classes of underlying this assumption is too restrictive as it does not allow for an implied volatility that depends on strike as it is observed in the market, at least since the Black Friday in 1987, see for a review and attempted explanation (Benzoni et al., 2011).

Hence the most natural extension of the existing models was to postulate either a state-dependent volatility often duped as local volatility (Derman and Kani, 1994; Dupire, 1994) or to postulate an additional process for the volatility (e.g., Hull and White, 1987; Heston, 1993) which are labeled as stochastic volatility models.

Looking at the properties of these two model classes it was found (Hagan et al., 2002) that local volatility is postulating a dynamics which is not found in real markets. In foreign exchange options markets stochastic volatility models tend to exaggerate the effect of volatility convexity and at the same time these models are unable to match the short–dated volatility smile observed in market–prices. As a practical workaround, models that mix the local volatility and stochastic volatility were developed (Said, 1999; Blacher, 2001). It was observed that the calibration of SLV models is a hard problem which requires either a specific parametrization to derive fast pricing of vanilla options or quite time-consuming numerical optimization procedures (Guyon and Henry-Labordere, 2011). See as well Homescu (2014) for a great summary and best practice of local stochastic volatility models.

A shortcut to derive manageable calibration times was developed by Guyon and Henry-Labordere (2011) and Van der Stoep et al. (2014) using a Monte Carlo procedure to derive the required estimation of the conditional variance. In this paper we suggest to use methods from machine learning, in particular radial basis functions and variations thereof to derive fast and efficient estimators.

## 2. Local Stochastic Volatility Calibration

The LSV model in general is of the form:

$$\begin{array}{l} dS_{t}=\mu \left(t\right)S_{t}dt+\sigma \left(S_{t},t\right)f\left(V_{t}\right)S_{t}dW_{t}\\ dV_{t}=\mu_{V}\left(V_{t}\right)dt+\xi \chi \left(V_{t}\right)dX_{t}\\ <dW_{t},dX_{t}>=\rho dt \end{array}$$

with spot *S*_*t*_, variance *V*_*t*_, drift μ, (state-dependent) drift for the variance μ_*V*_, vol of variance ξχ(*V*_*t*_), and correlation ρ. The LSV calibration is the process to determine the leverage function σ given the local volatility function $\sigma_{Dupire}^{2}$ and all the other parameters of the model. There is a fundamental relationship of the leverage function and the local volatility function (Dupire, 1996) where the expectation $E^{P\left(S_{t},V_{t},\sigma \right)}$ of the conditional variance *V*_*t*_ is taken with respect to the risk–neutral measure induced by the model. The notation indicates that *P*(*S*_*t*_, *V*_*t*_, σ) is the joint probability of spot process *S*_*t*_, variance process *V*_*t*_ and the solution for σ which depends on the probability distribution of (*S*_*t*_, *V*_*t*_).

(1) $$\begin{array}{l} \sigma_{Dupire}^{2}\left(S_{t},t\right)=E^{P\left(S_{t},V_{t},\sigma \right)}\left(V_{t}|S=S_{t}\right)\sigma^{2}\left(S_{t},t\right) \end{array}$$

Plugging the solution into the model equation makes this a McKean SDE where the expectation depends on the probability of the process itself.

To solve this equation Monte Carlo simulation can be used. The equations are discretized and the forward propagation of the spot *S*_*t*_ and variance *V*_*t*_ is interleaved with the estimation of conditional expectation using the realized paths of *S*_*t*_ and *V*_*t*_. Contrary to standard Monte Carlo where all paths develop independently we need to bring all simulated paths to the estimation procedure. Using Euler discretization

$$\begin{array}{l} \Delta \ln\left(S_{t}\right)=\mu \left(t\right)\Delta t-\frac{1}{2}\sigma^{2}\left(S_{t},t\right)f^{2}\left(V_{t}\right)\Delta t\\ +\sigma \left(S_{t},t\right)f\left(V_{t}\right)\left(\sqrt{1-\rho^{2}}\Delta W+\rho \Delta X\right)\\ \Delta V_{t}=\mu_{V}\left(V_{t}\right)\Delta t+\xi \chi \left(V_{t}\right)\Delta X \end{array}$$

with Δ*W*, Δ*X* independent increments. The estimation of the conditional expectation can be seen as finding the function *R*(*S*) = *E*(*V*_*t*_|*S*_*t*_ = *S*) based on the samples as observed pairs

$$\left(S_{t}^{1},V_{t}^{1}\right),\cdots \;,\left(S_{t}^{n},V_{t}^{n}\right)$$

where the spot $S_{t}^{i}$ and variance $V_{t}^{i}$ are the time *t* realizations of spot and variance on path *i*. Originally it was proposed to estimate the function *R* using kernel regression (Guyon and Henry-Labordere, 2011):

(2) $$\begin{array}{l} R\left(\left(S^{1},V^{1}\right),\cdots \;,\left(S^{N},V^{N}\right)\right)\left(S\right)=\frac{\sum_{i=1}^{N}V_{i}K_{h}\left(S-S_{i}\right)}{\sum_{i=1}^{N}K_{h}\left(S-S_{i}\right)} \end{array}$$

with Kernel functions *K*_*h*_, where we dropped the *t* index as it is clear from the context. Alternatively (Van der Stoep et al., 2014) proposed to use binning techniques or sets of polynomials.

Subsequently we will evaluate alternative regression techniques to estimate the conditional expectation based on the realized paths. This can be rephrased as a supervised learning problem where each path is a (noisy) example.

## 3. Regression as a Supervised Learning Problem

The task to find a relationship between some input variables and an output from examples is one of the problems tackled by machine learning and is well studied as supervised learning. There are many classes of supervised learning algorithms and setups and we would like to demonstrate guidelines to which specific choices are suitable for the problem at hand. The basic problem is, given a set of examples *x*_*i*_, *y*_*i*_ to find a function *f*(*x*) such that an error functional is minimized. The task is to find a function such that there is low error on unseen examples, this is called generalization. There is a balance to strike between the error on the examples used for training and the error on the validation set of examples which are examples not used during training, for a fundamental analysis of the learning theotry and the relation between capacity and the generalization (see e.g., Vapnik, 2013), in particular chapter 4.

### 3.1. Kernel Regression

The approach taken in Guyon and Henry-Labordere (2011) as stated above is Nadarajan-Watson kernel regression which is one of the so called non-parametric methods. The method is identical to Equation (2). The estimator is given as:

$$\begin{array}{l} R\left(\left(x_{1},y_{1}\right),\cdots \;,\left(x_{N},y_{N}\right)\right)\left(X\right)=\frac{\sum_{i=1}^{N}y_{i}K_{h}\left(x-x_{i}\right)}{\sum_{i=1}^{N}K_{h}\left(x-x_{i}\right)} \end{array}$$

In this approach a Kernel function *K*(*x*) is used, which satisfies:

$$\begin{array}{lll} K\left(x\right) & \geq & 0\\ K\left(x\right)=K\left(-x\right)\\ \int_{-\infty }^{\infty }K\left(x\right)dx=1\\ K_{h}\left(x\right)=\frac{1}{h}K\left(\frac{x}{h}\right) \end{array}$$

There is a variety of Kernel functions well studied in the literature (Härdle, 1990):

- Gaussian $\frac{1}{\sqrt{2\pi }}e^{-\frac{x^{2}}{2}}$
- Quartic $\frac{15}{16}{\left(1-x^{2}\right)}^{2}$
- Epanechnikov $\frac{3}{4}\left(1-x^{2}\right)$
- Sigmoid $\frac{2}{\pi }\frac{1}{e^{x}+e^{-x}}$

Often the Kernel function used is Gaussian hence the support of the function is infinite or it will be the Epanechnikov Kernel which has bounded support.

The crucial choice is the bandwidth of the Kernel functions. There is a rule-of-thumb derived from normal distribution assumptions, (Silverman, 1986):

$$\begin{array}{l} h={\left(\frac{4\sigma^{5}}{3n}\right)}^{\frac{1}{5}} \end{array}$$

for the standard deviation σ of the data and *n* data points.

Alternatively cross validation, particular “leave–one–out cross validation” can be used to determine an optimal Kernel width. Cross validation is quite costly computationally and hence can only be used to cross check *ad-hoc* choices.

Local Linear Kernel Regression is a variation of Kernel regression which employs local linear terms and which is given by the solution of

$$\begin{array}{lll} R\left(\left(x_{1},y_{1}\right),\cdots \;,\left(x_{N},y_{N}\right)\right)\left(x\right)=\underset{\alpha ,\beta }{\min}\overset{N}{\underset{i=1}{\sum }}{\left(y_{i}-\alpha -\left(x-x_{i}\right)\beta \right)}^{2}\\ \; & \; & K_{h}\left(x-x_{i}\right) \end{array}$$

The minimum is found by solving a 2 × 2 linear system.

In general the Kernel approaches suffers from some systematic shortcomings, mainly the fact that all examples are used, no compression happens, secondly a bias is introduced close to the boundary and the difficult choice of suitable bandwidth, where practically sound theoretical methods as cross validation cannot be used for computation time reasons.

### 3.2. Radial Basis Functions

Radial Basis Functions (RBF) and Partition of Unity Radial Basis Functions (PURBF) respectively take the form

$$\begin{array}{l} RBF\left(x\right)=\overset{C}{\underset{i=1}{\sum }}w_{i}K_{h_{i}}\left(x-c_{i}\right)\\ PURBF\left(x\right)=\frac{\sum_{i=1}^{C}w_{i}K_{h_{i}}\left(x-c_{i}\right)}{\sum_{i=1}^{C}K_{h_{i}}\left(x-c_{i}\right)} \end{array}$$

PURBF are quite similar in functional form to Kernel regression. The main difference is that the number of basis functions is much smaller than the number of examples. It was proven that RBF and PURBF are universal function approximators (Hakala et al., 1994) which makes them suitable to approximate our estimation problem. If the *L*_2_ norm is used the weights are optimized by solving the normal equation

$$\begin{array}{l} LS=\frac{1}{2N}\overset{N}{\underset{i=1}{\sum }}{\left(y_{i}-RBF\left(x_{i}\right)\right)}^{2}. \end{array}$$

The solution s given by the weights *w*_*i*_, which satisfy

$$\begin{array}{l} w_{i}={\left(A^{T}A\right)}_{ik}^{-1}A_{kj}y_{j} \end{array}$$

with the matrix *A*_*ij*_ given as

(3) $$\begin{array}{l} A_{ij}=K_{h_{j}}\left(x_{i}-c_{j}\right). \end{array}$$

The remaining parameters are determined heuristically:

- centers *c*_*i*_ - in 1D we chose min(*x*_*i*_), max(*x*_*i*_), and a random subset of the remaining *x*_*i*_. Centers which are too close (in their local width) are pruned—the criterion for unit *j* is $\underset{i}{\min}\left(\frac{\left|c_{i}-c_{j}\right|}{h_{j}}\right)\leq \text{Θ}$ with a suitable global pruning constant Θ. There are resource allocating approaches as well (e.g., Fritzke, 1994) which seem less suitable here.
- width *h*_*i*_- are chosen individually as a multiple of the *k* nearest neighbors or *ad-hoc*.

#### 3.2.1. Regularization

Often the solution of the normal equation will be ill-conditioned. To counteract the bad conditioning of the problem and to get a better generalization we will use a regularizer on the *L*_2_ norm of the weights (e.g., Goodfellow et al., 2016, Chapter 7.1).

$$\begin{array}{l} LSR=\frac{1}{2N}\overset{N}{\underset{i=1}{\sum }}{\left(y_{i}-RBF\left(x_{i}\right)\right)}^{2}+\lambda \overset{C}{\underset{j=1}{\sum }}w_{j}^{2} \end{array}$$

The corresponding solution is given as

$$\begin{array}{l} w_{i}={\left(A^{T}A-\lambda \;id\right)}_{ij}^{-1}A_{jk}y_{k} \end{array}$$

with identity matrix *id*. The same solution applies for the PURBF function instead of the RBF one.

## 3.3. Computational Efficency

For standard Kernel Regression computational effort is mainly due to sorting O(n log(n)) of the spot observations to enable an efficient lookup of relevant spot observations during the retrieval phase. Optimal determination of width (cross-validation) requires the evolution of all kernels at all points several times which is very costly compared to the lookup. Local linear Kernel Regression requires an additional inversion of a 2 × 2 matrix which is negligible. For RBF and PURBF the solution to a small linear system is required. In particular the size is much smaller than the number of samples. Sorted examples can be used to optimize the training as the required matrix is determined by sums over the samples. Width and pruning computations require local computation of the order of the number of kernel functions. Overall the computational effort for RBF/PURBF is comparable and might be smaller in the retrieval phase than for Kernel Regression itself.

## 3.4. Alternative Architectures

In the last couple of years popularity of multilayer perceptron (MLP) and deep versions thereof grew enormously. For our application we rule out these architectures as the training is much more involved in the MLP case with a many remaining questions about a suitable number of hidden units, number of layers, type of activation functions. We could envision to use a pretrained MLP to get the solution without training. We postpone this approach for potential future use.

## 4. Application to LSV Model in Foreign Exchange

The model we will study is of Heston type

$$\begin{array}{l} dS_{t}=\mu \left(t\right)S_{t}dt+\sigma \left(S_{t},t\right)\sqrt{V_{t}}S_{t}dW_{t}\\ dV_{t}=\kappa \left(\bar{V}-V_{t}\right)dt+\xi \sqrt{V_{t}}dX_{t}\\ <dW_{t},dV_{t}>=\rho dt \end{array}$$

with mean reversion speed κ, mean reversion level $\bar{V}$.

The advantage is that we have a semi-closed form solution for vanilla call- and put options in the Heston model without the leverage function hence the first step is to calibrate the Heston model and then apply a scaling to the vol of vol parameter to reduce the SV impact and to let the local volatility compensate to match the vanilla option market. In this study we will use a volatility mixing of 66% which means that we scale the vol of variance by this factor before calibrating of the leverage function.

To compare the performance of the various regression algorithms on this model we will show for a specific slice the realized spot/variance and the corresponding results of the regression functions.

### 4.1. Example EUR/USD 6M

**Figure 1:**
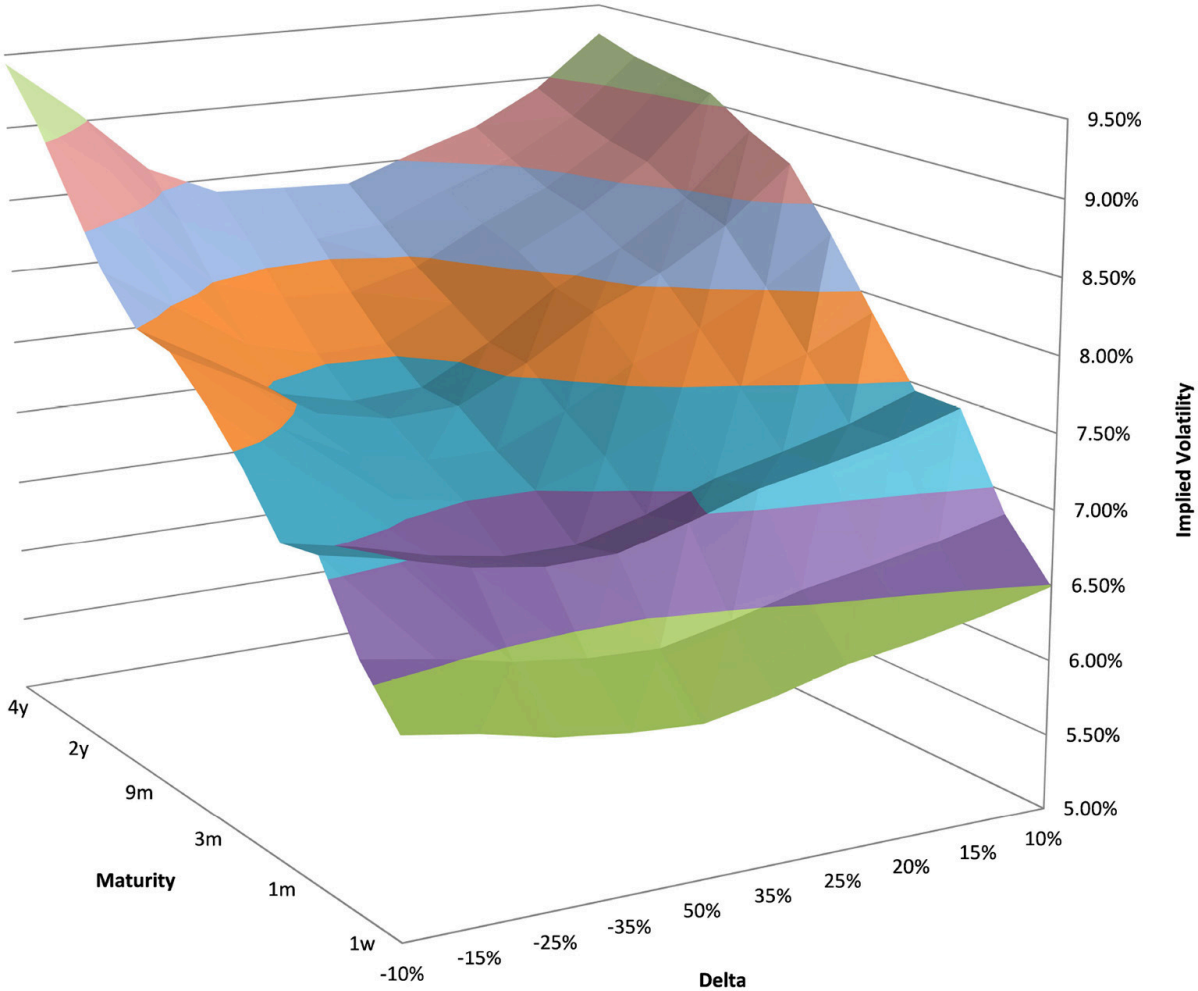
EUR/USD implied volatility in log moneyness and time to maturity. (Source: Leonteq AG—March 2018).

**Figure 2:**
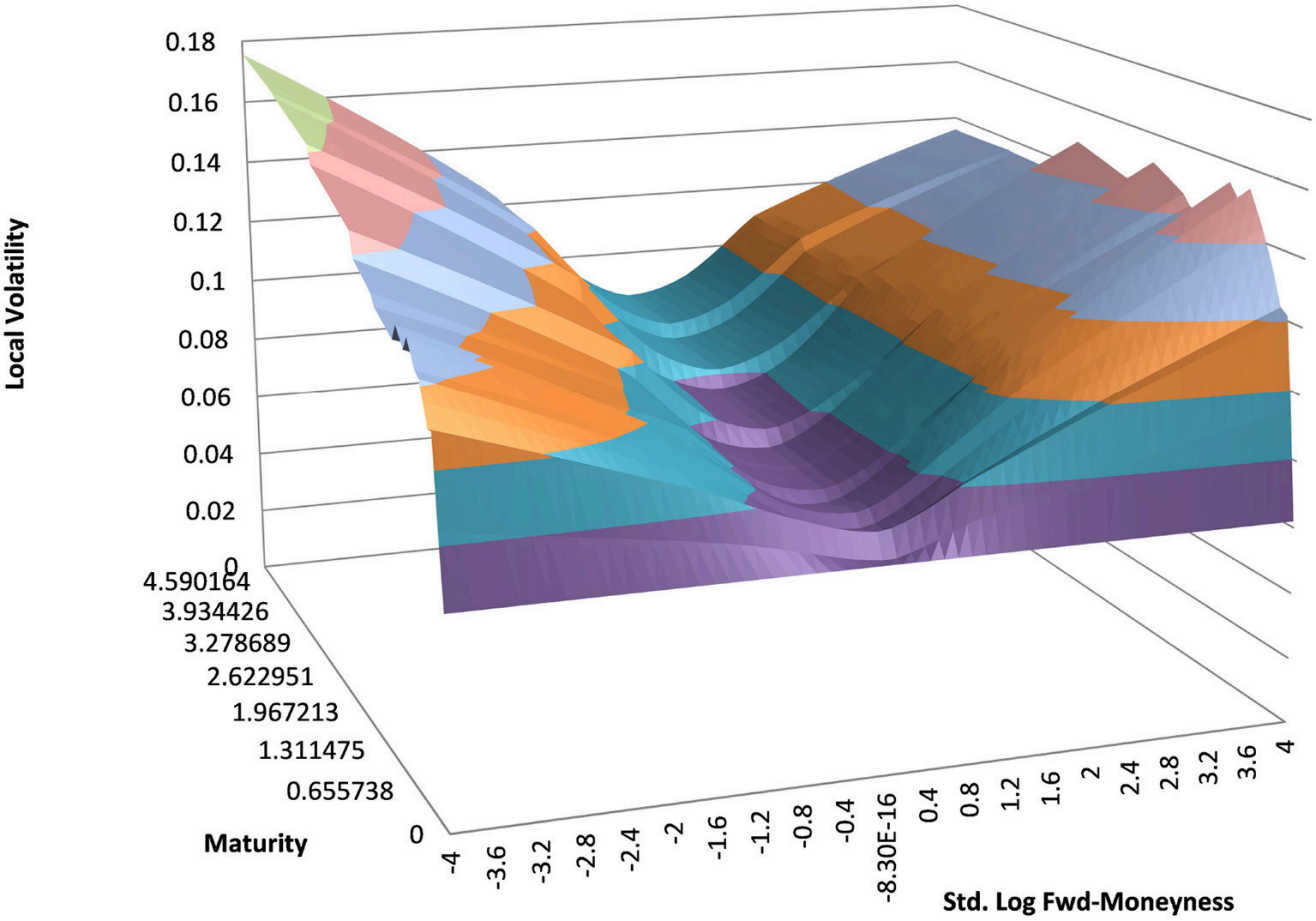
EUR/USD local volatility in log moneyness and time to maturity. (Source: Leonteq AG—March 2018).

**Figure 3:**
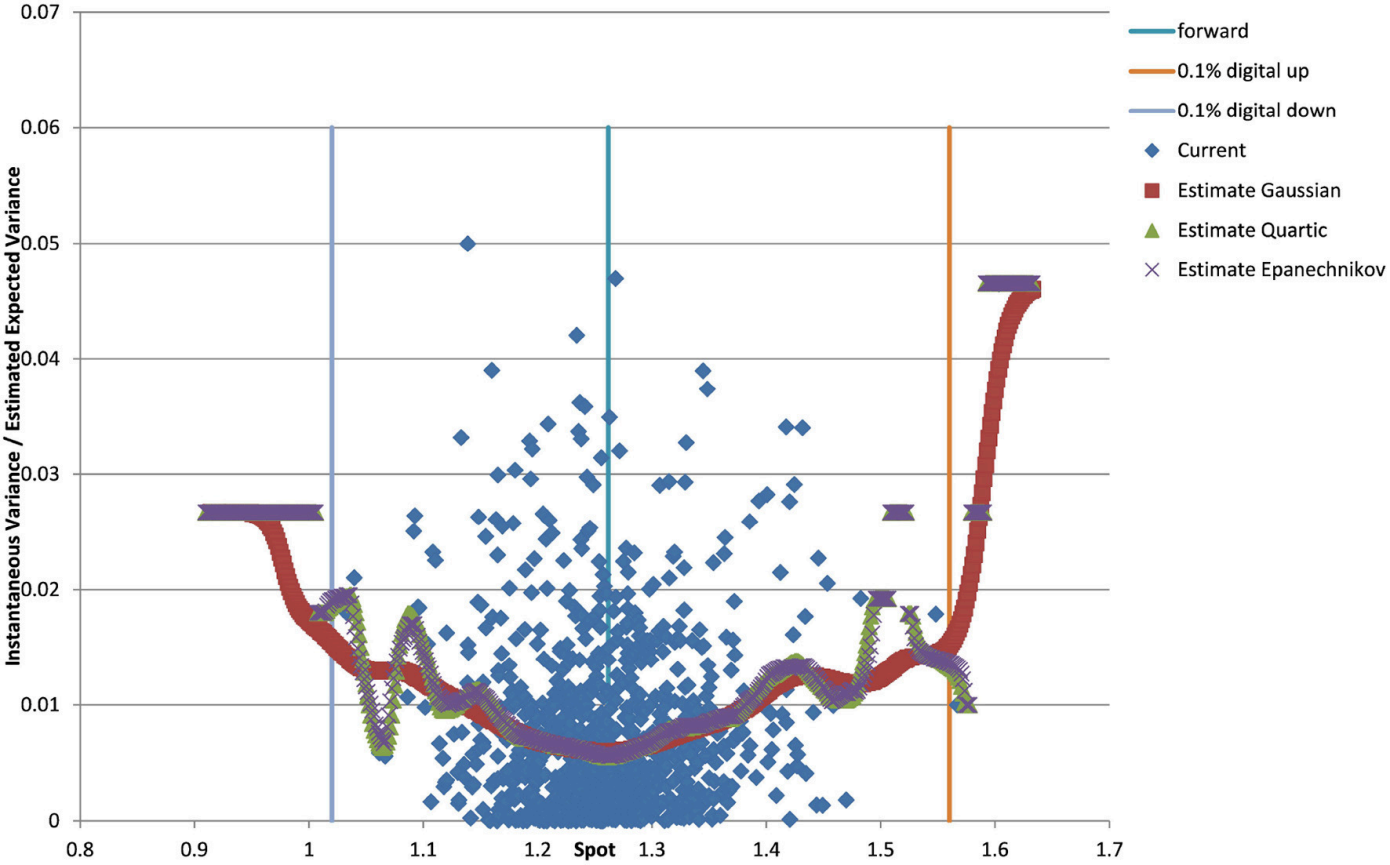
EUR/USD 6M estimated variance conditional on realized spot and realized paths for various kernel functions as well as forward and digital levels. (Source: Leonteq AG—March 2018).

**Figure 4:**
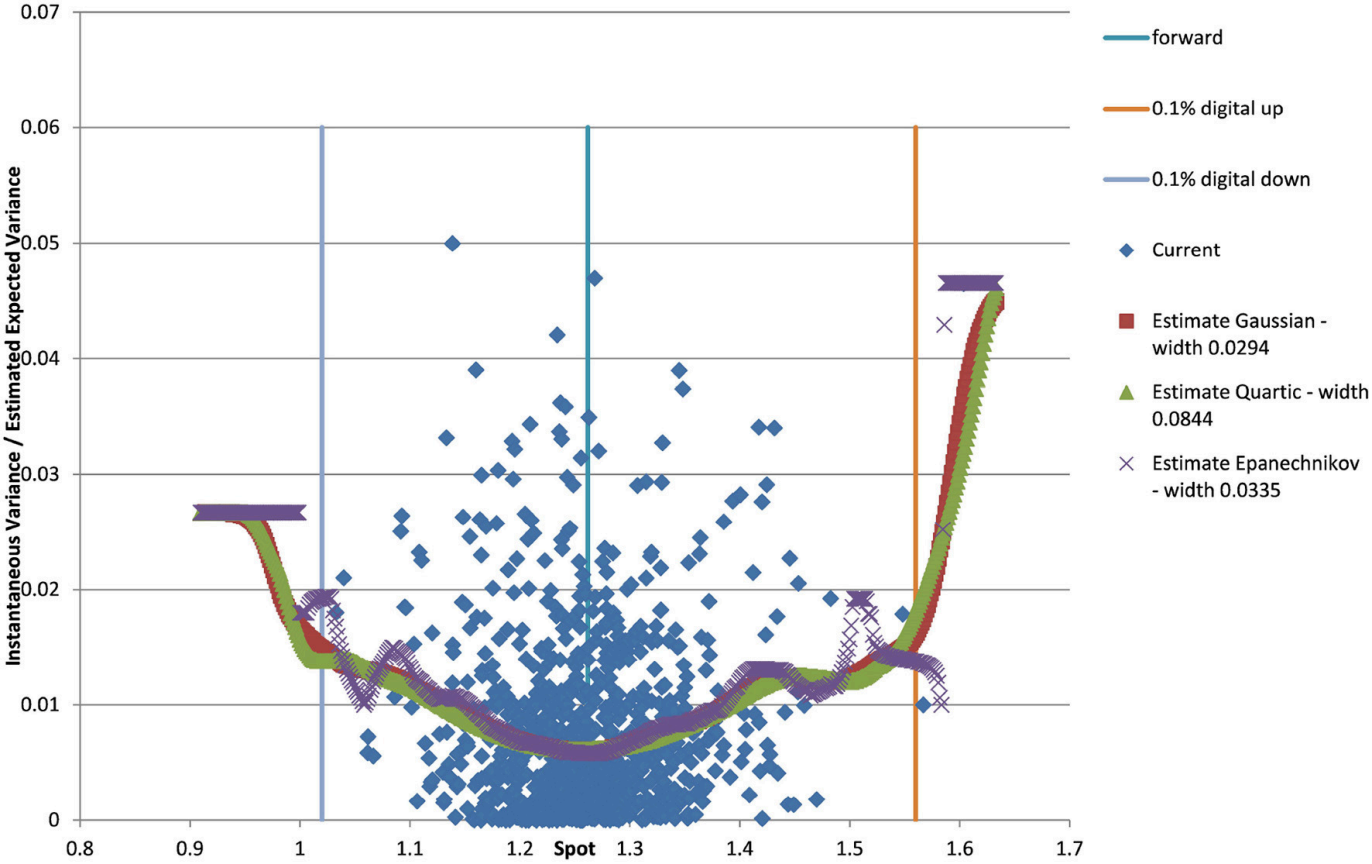
EUR/USD 6M estimated variance conditional on realized spot and realized paths for various kernel functions and cross validated width as well as forward and digital levels. (Source: Leonteq AG—March 2018).

**Figure 5:**
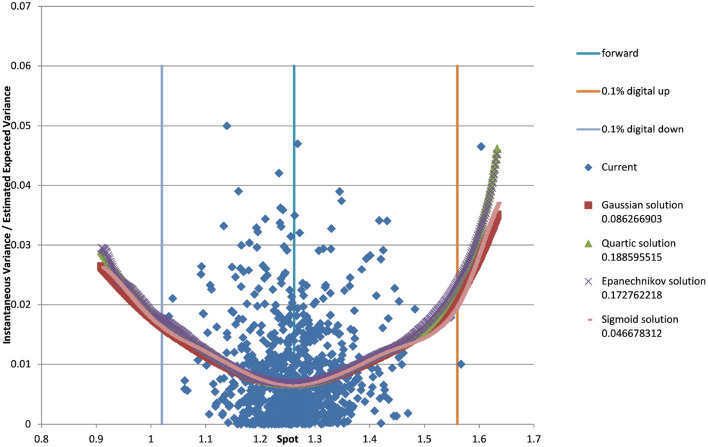
EUR/USD 6M estimated variance conditional on realized spot and realized paths for various local linear kernel functions and cross validated width as well as forward and digital levels. (Source: Leonteq AG—March 2018).

You can see the volatility surface in Figure 1 and the corresponding local volatility surface in Figure 2. The snapshot of data, including spot, volatility, and interest rates was taken in March 2018. We show the results of different kernel estimators, using Silverman's rule of thumb for the width, including the samples indicated as *Current*, as well as the forward and the level of a 0.1% digital on the upside and downside Figure 3. In Figure 4 you see the results of Kernel Regression for Silverman's rule and additionally the cross-vaildated width for the same kernel functions. Notice that the optimal width varies between different kernels. We show results for local linear kernel regression in Figure 5. It can be seen that the bias at the boundaries is reduced in comparison to the kernel regression. Again we show results for Silverman's rule and additionally the cross-vaildated width.

**Figure 6:**
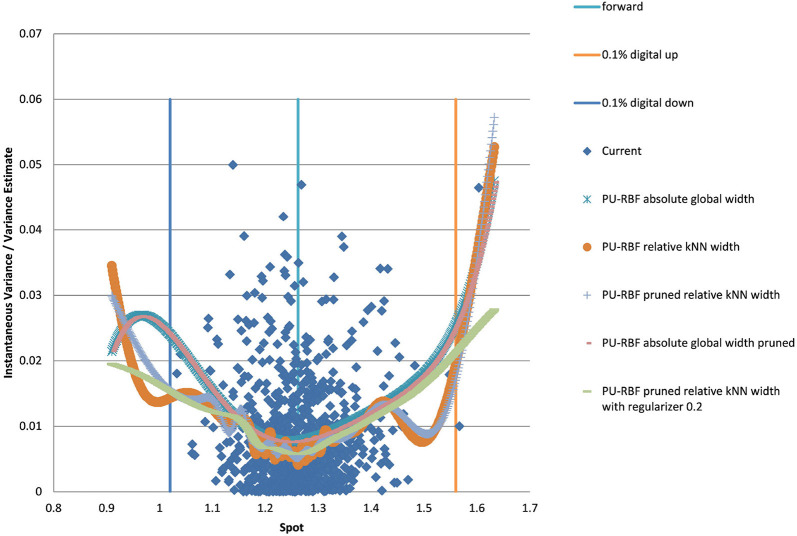
EUR/USD 6M estimated variance conditional on realized spot and realized paths using PURBF as well as forward and digital levels. (Source: Leonteq AG—March 2018).

For the PURBF we show results in Figure 6 using a global width, relative knn width, pruned and relative knn width, pruned with global width, and pruned, knn width and regularizer (λ = 0.2). We use 40 units in all cases as this number seems sufficently versatile for the number of particles we want to use (2,048).

The last version with regularizer, pruning, and local width is the preferred version as it shows a smooth behavior without a bias in the boundaries and matches the part with many data–points in the middle without oscillations.

### 4.2. Example EUR/USD 5Y

**Figure 7:**
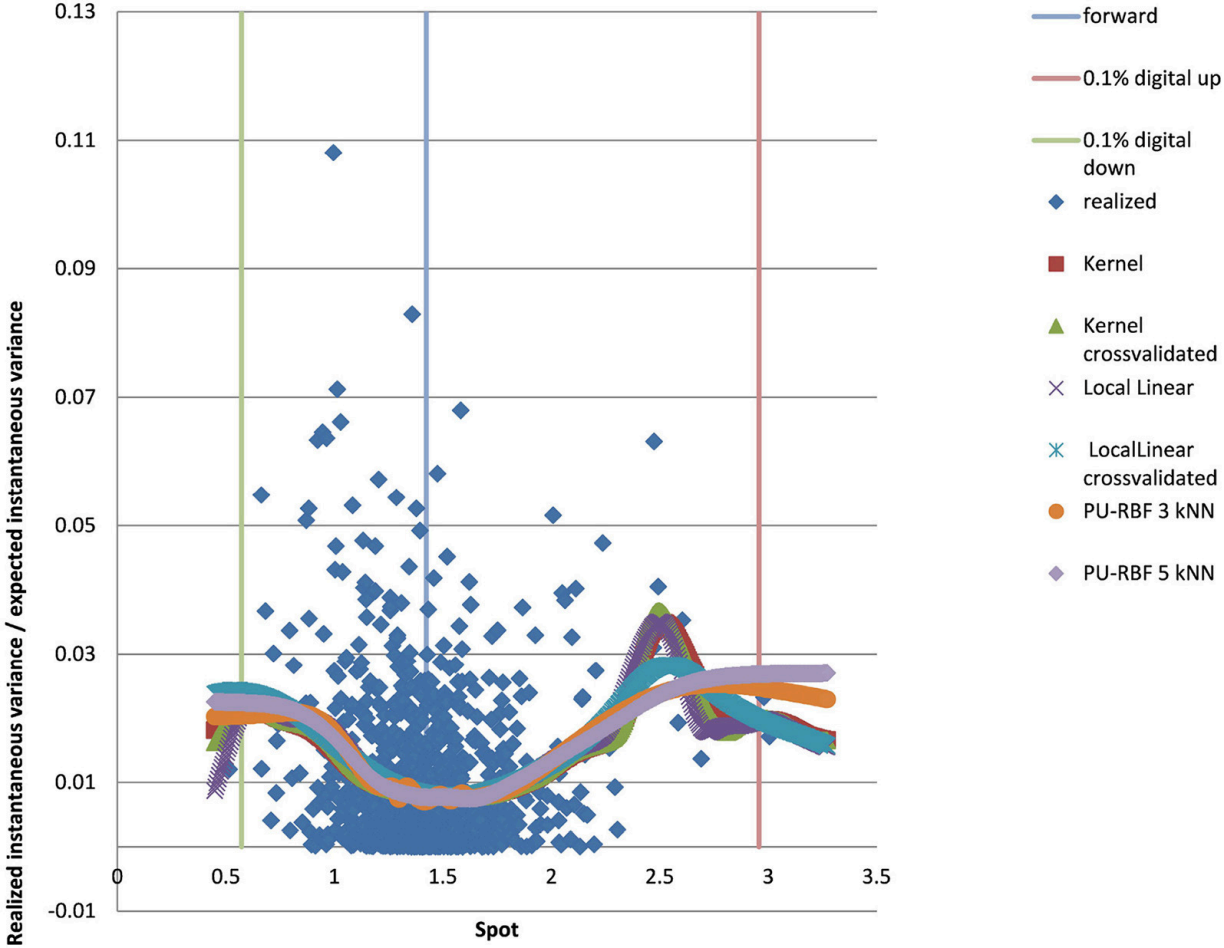
EUR/USD 5Y estimated variance conditional on realized spot and realized paths using kernel regression, local linear kernel regression, and PURBF as well as forward and digital levels. (Source: Leonteq AG—March 2018).

We show the results for 5Y maturity and the same volatility surface in Figure 7. Among the tested approaches the PURBFwith 5 nearest neighbors performs best.

### 4.3. Example USD/JPY 5Y

**Figure 8:**
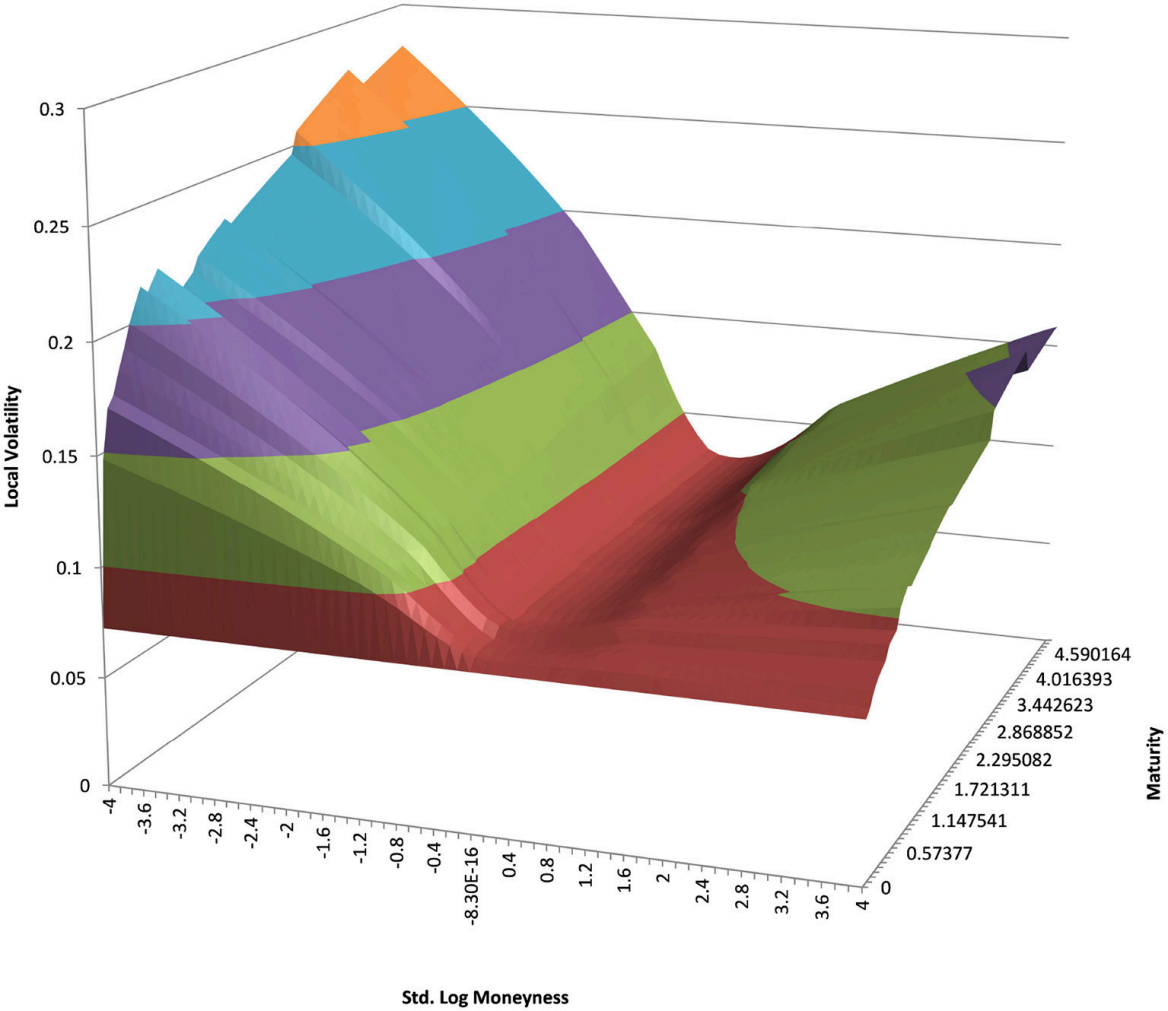
USD/JPY local volatility in log moneyness and time to maturity. (Source: Leonteq AG—March 2018).

**Figure 9:**
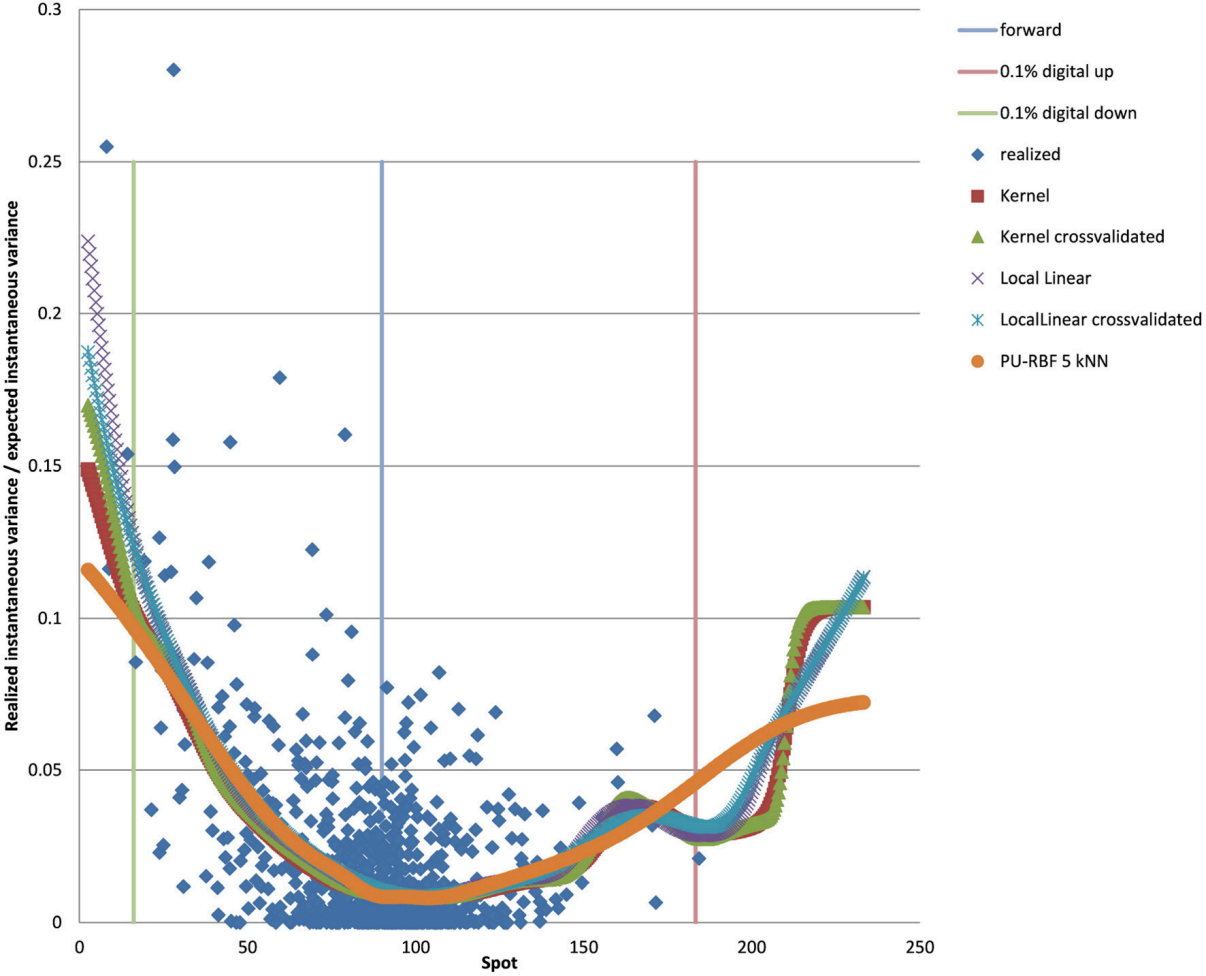
USD/JPY 5Y estimated variance conditional on realized spot and realized paths using kernel regression, local linear kernel regression, and PURBF as well as forward and digital levels. (Source: Leonteq AG—March 2018).

We show the results for USD/JPY, see the local volatility surface in Figure 8. The estimation across the spot range is shown in Figure 9. Again the PURBFwith 5 nearest neighbors performs best.

### 4.4. Example EUR/BRL 3Y

**Figure 10:**
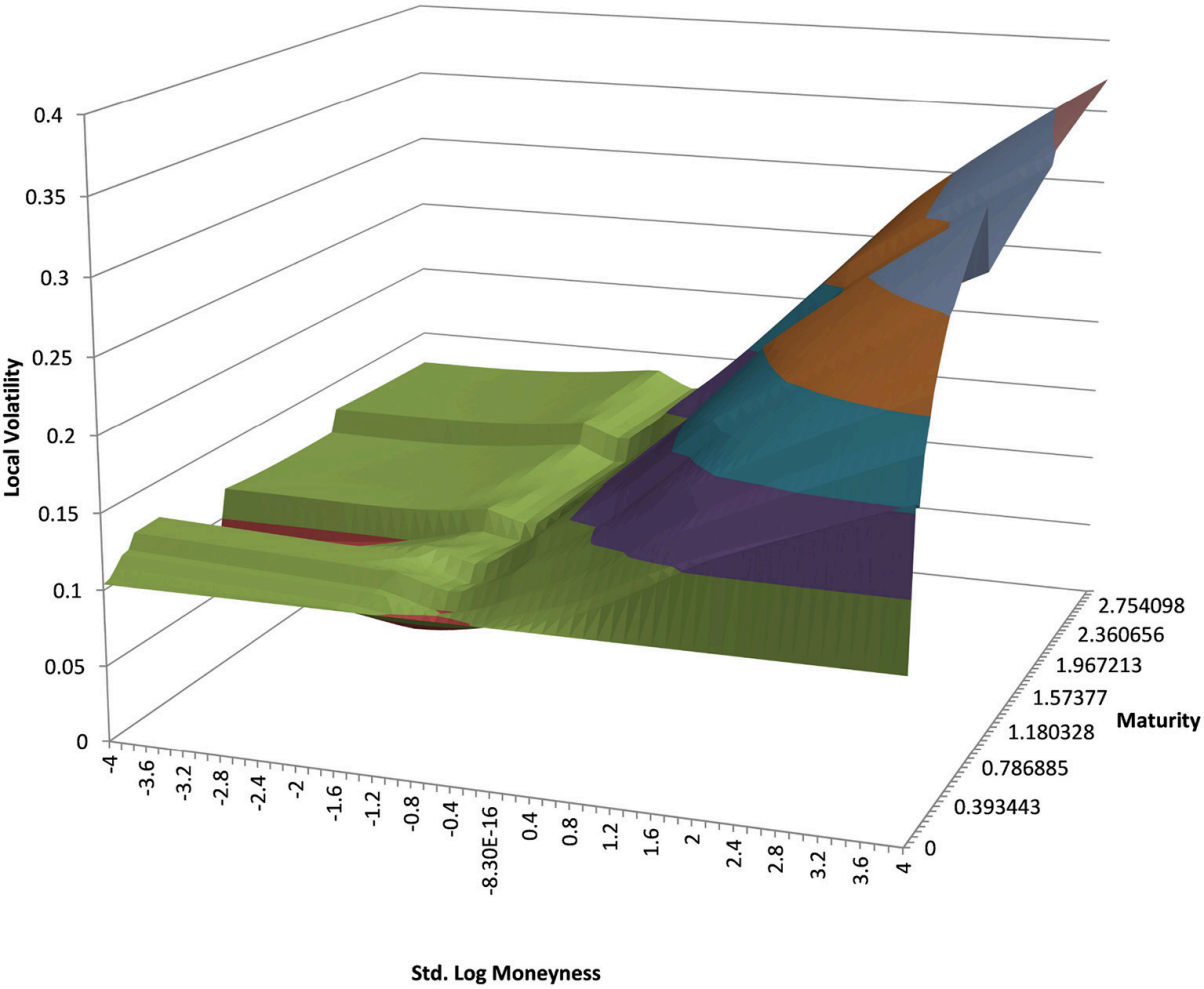
EUR/BRL local volatility in log moneyness and time to maturity. (Source: Leonteq AG—March 2018).

**Figure 11:**
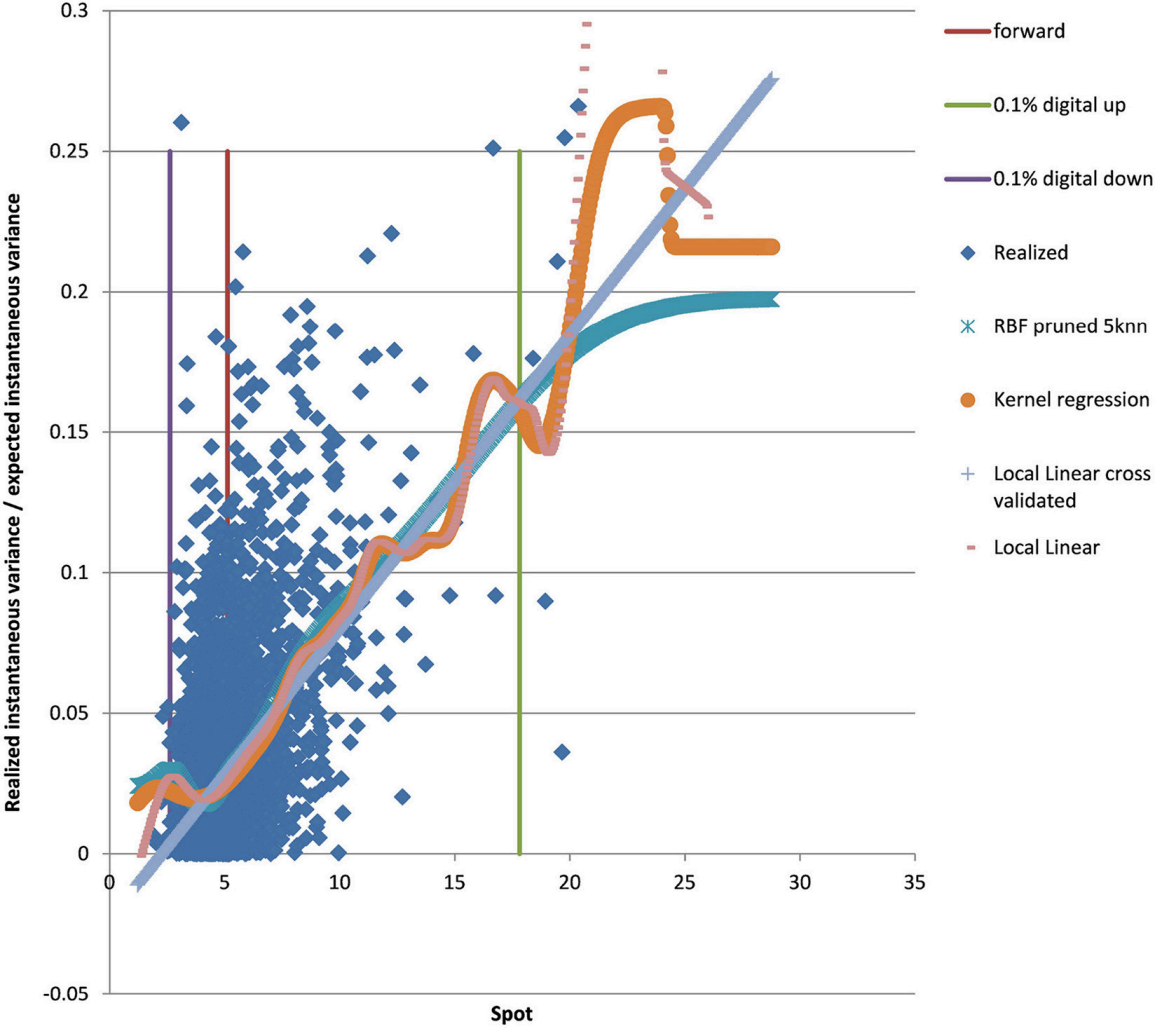
EUR/BRL 3Y 5Y estimated variance conditional on realized spot and realized paths using kernel regression, local linear kernel regression, and PURBF as well as forward and digital levels. (Source: Leonteq AG—March 2018).

We show the results for EUR/BRL, which is a highly skewed and highly drifting underlying. See the local volatility surface in Figure 10. The estimation across the spot range is shown in Figure 11. Note that in this case the range of spot realizations is quite skewed as is expected from the skewed volatility surface. Nevertheless the PURBFwith 5 nearest neighbors puts a relatively smooth estimator through the samples and performs better than other methods.

### 4.5. Pricing Examples

To see the impact on exotics pricing we look at one–touch options. A one–touch option pays one unit of the counter currency at the maturity date if the spot trades at or beyond the touch–level at any time during the life of the option. We show the impact as a function of the Black–Scholes price (TV), similar to (Clark, 2011). The TV of a one–touch can be between 0% and the discount factor to maturity, which is in the range of 100%. For fixed market parameters like spot, volatility and the risk–neutral drift TV is a function of the touch–level only, hence makes a unique scale to show the model impact. The deviation of the LSV model price from the TV is the desired effect of an alternative model, which incorporates volatility risk management and hedging, compared to the Black–Scholes model. The form of the deviation is not obvious and would require a rather complicated hedging argument of volatility risk and cross spot–volatility risk.

**Figure 12:**
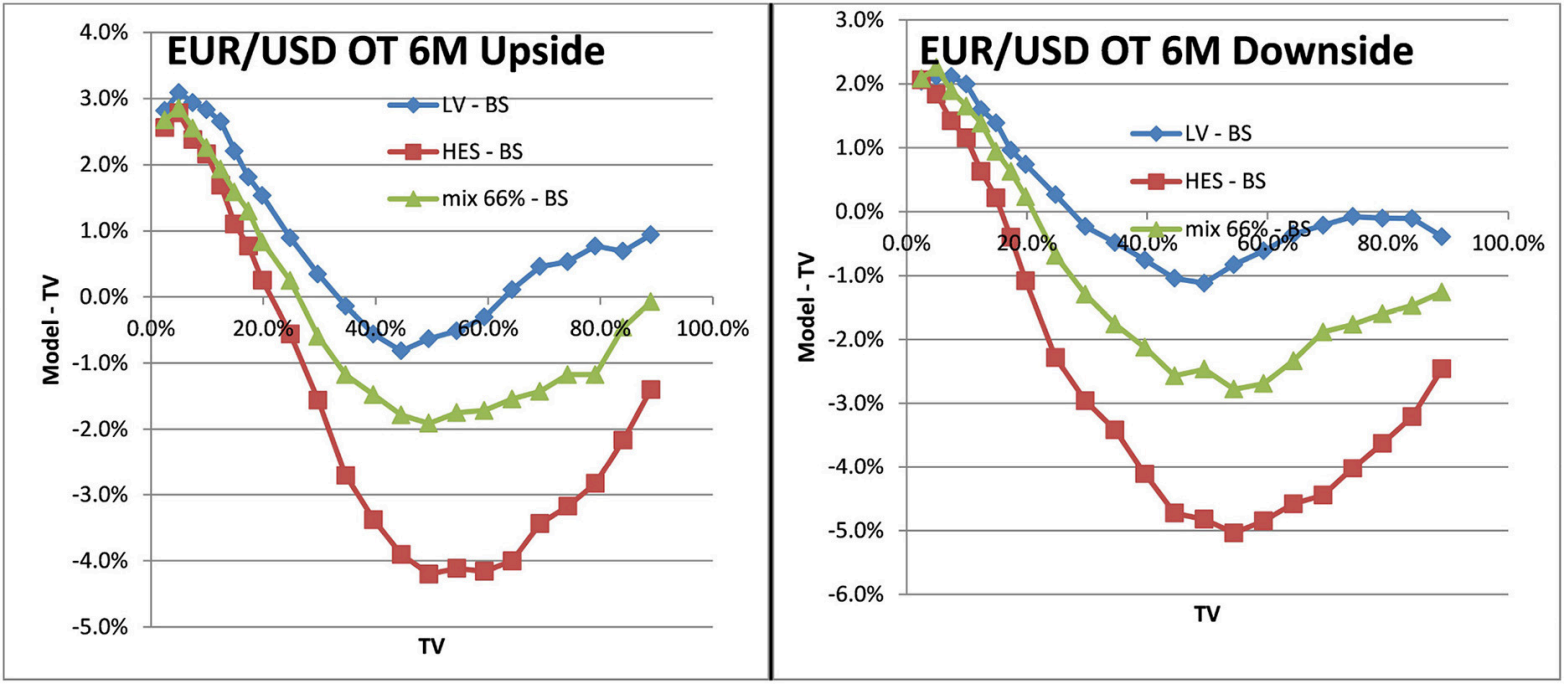
One–touch prices EUR/USD 6M LV vs. TV, SV vs. TV, LSV vs. TV. Upside one–touches on the left, downside on the right.(Source: Leonteq AG—March 2018).

**Figure 13:**
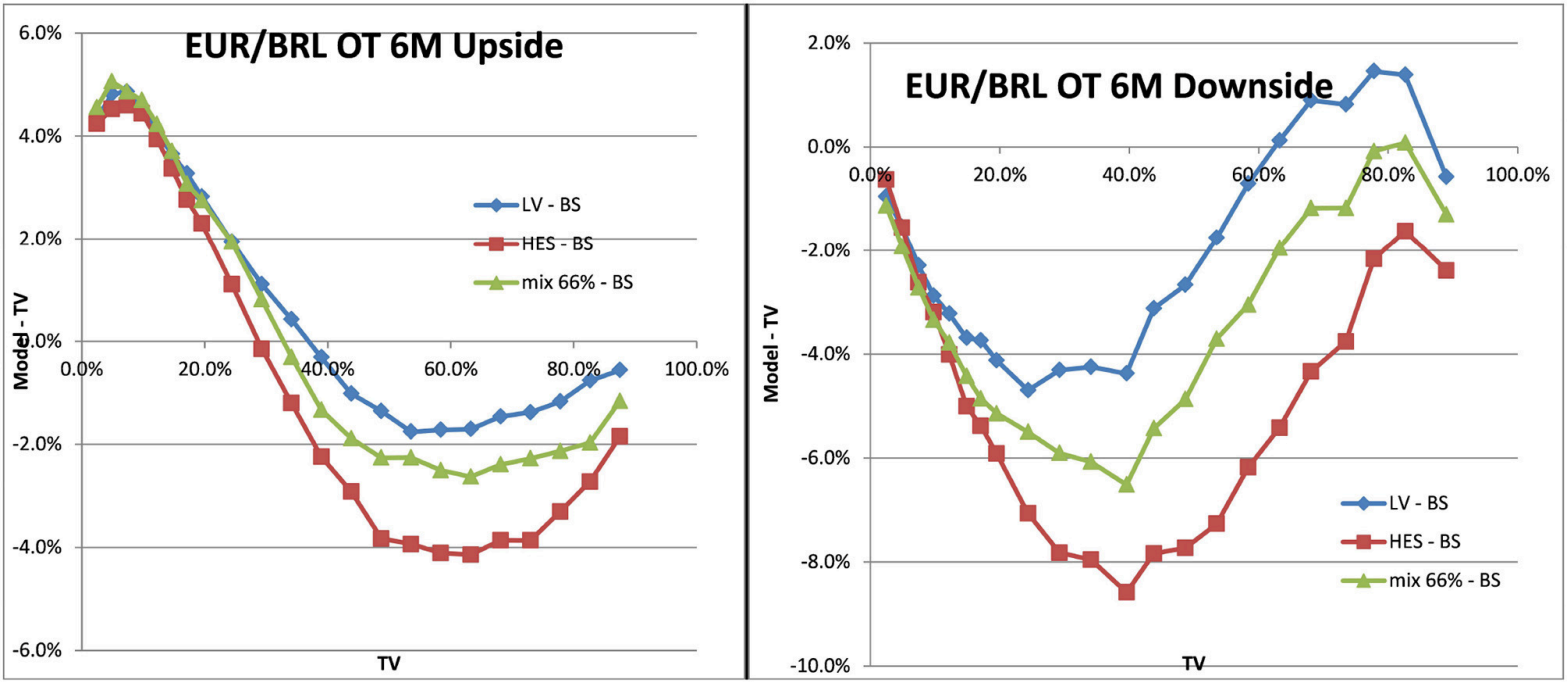
One–touch prices EUR/BRL 6M LV vs. TV, SV vs. TV, LSV vs. TV. Upside one–touches on the left, downside on the right. (Source: Leonteq AG—March 2018).

With the mixed local stochastic volatility model and mixing rate of 66% we expected the LSVprice to be within the bounds of stochastic and local volatility price. We use Monte Carlo pricing with a fixed number of 32,000 paths (antithetic) and Quasi Random Numbers, a time discretization of 5 days and fixed 2048 particles. We denote the Black–Scholes prices as BS or TV (theoretical value) in the graphs and use LV as abbreviation for prices in local volatility and HES for the Heston model without local volatility component. The prices can be seen in Figure 12 for EUR/USD 6M and in Figure 13 for EUR/BRL 6M. We observe the expected behavior in all cases, the mixed local stochastic volatility prices are within the range of local and stochastic volatility prices and the mixing parameter can be used to adjust the behavior to observed exotics prices (e.g., one touches) in the market. Usually this mixing parameter is quite stable across longer periods, often weeks or even months.

## 5. Conclusion

We apply machine learning principles to improve the calibration process of the local stochastic volatility models. The suggested meta parameters and heuristics seem to apply to a wide variety of underlyings in FX, liquid pairs like EUR/USD as well as emerging markets as EUR/BRL. The computational efficiency is at about the same level as for the formerly suggested Kernel Regression based approach. The results given by the PURBF function with pruning, regularization, and local width determined by 5 nearest neighbor performed significantly better than the Kernel based approaches, hence we would suggest to consider this approach in the calibration process.

Further work will be dedicated to improve the computational speed and to establish better measures of the quality. In particular in situations where vol surfaces are almost arbitragable we will need the method to continue to provide numerically stable results.

## Author Contributions

The author confirms being the sole contributor of this work and has approved it for publication.

### Conflict of Interest Statement

The author declares that the research was conducted in the absence of any commercial or financial relationships that could be construed as a potential conflict of interest.
